# Substituting Tyr^138^ in the active site loop of human phenylalanine hydroxylase affects catalysis and substrate activation

**DOI:** 10.1002/2211-5463.12243

**Published:** 2017-06-12

**Authors:** João Leandro, Anne J. Stokka, Knut Teigen, Ole A. Andersen, Torgeir Flatmark

**Affiliations:** ^1^ Department of BiomedicineUniversity of BergenNorway; ^2^ Metabolism and Genetics GroupResearch Institute for Medicines (iMed.ULisboa)Faculty of PharmacyUniversity of LisbonPortugal; ^3^ The Biotechnology Centre of OsloUniversity of OsloNorway; ^4^ Evotec (UK) Ltd.AbingdonUK; ^5^ Present address: Department of Genetics and Genomic SciencesIcahn School of Medicine at Mount Sinai1425 Madison Avenue, Box 1498New YorkNY10029USA

**Keywords:** active site loop, phenylalanine hydroxylase, substrate activation

## Abstract

Mammalian phenylalanine hydroxylase (PAH) is a key enzyme in l‐phenylalanine (l‐Phe) metabolism and is active as a homotetramer. Biochemical and biophysical work has demonstrated that it cycles between two states with a variably low and a high activity, and that the substrate l‐Phe is the key player in this transition. X‐ray structures of the catalytic domain have shown mobility of a partially intrinsically disordered Tyr^138^‐loop to the active site in the presence of l‐Phe. The mechanism by which the loop dynamics are coupled to substrate binding at the active site in tetrameric PAH is not fully understood. We have here conducted functional studies of four Tyr^138^ point mutants. A high linear correlation (*r* ^2^ = 0.99) was observed between their effects on the catalytic efficiency of the catalytic domain dimers and the corresponding effect on the catalytic efficiency of substrate‐activated full‐length tetramers. In the tetramers, a correlation (*r* ^2^ = 0.96) was also observed between the increase in catalytic efficiency (activation) and the global conformational change (surface plasmon resonance signal response) at the same l‐Phe concentration. The new data support a similar functional importance of the Tyr^138^‐loop in the catalytic domain and the full‐length enzyme homotetramer.

**Abbreviations**
CD: catalytic domain
MBP: maltose‐binding protein
MCD: magnetic circular dichroism
*n*_H_: Hill coefficient
PAH: phenylalanine hydroxylase
SAXS: small‐angle X‐ray scattering
SEC: size‐exclusion chromatography
SPR: surface plasmon resonance
XAS: X‐ray absorption spectroscopy

The mononuclear non‐heme iron‐containing enzyme phenylalanine hydroxylase (PAH, phenylalanine 4‐monooxygenase, EC 1.14.16.1) catalyzes the stereospecific hydroxylation of l‐phenylalanine (l‐Phe) to l‐tyrosine, the first and rate‐limiting step in the catabolism of dietary phenylalanine in the liver [for reviews, see 1, 2]. The enzyme uses a five‐coordinated Fe(II) to activate dioxygen in the tightly coupled hydroxylation of l‐Phe with the pterin cofactor (6*R*)‐L‐*erythro*‐5,6,7,8‐tetrahydrobiopterin (BH_4_) as a two‐electron donor. wt‐PAH is a homotetramer in equilibrium with a dimeric form (the rate of interconversion is considered to be slow 1). The subunit [452 residues for the human PAH (hPAH)] consists of three major structural and functional domains: an N‐terminal autoregulatory domain (RD, residues 1–117), a central catalytic core domain (CD, residues 118–410), and a C‐terminal oligomerization domain (residues 411–452), with an antiparallel β‐sheet dimerization motif and an α‐helical tetramerization motif (for review, see 2). For the enzyme tetramer to work properly, these domains and the four subunits must communicate with each other by an intricate dynamic network which is yet only partly characterized.

The enzyme is transiently activated by l‐Phe 3, which was studied in more details by Shiman *et al*. (for review, see 4) and others. Based on enzyme kinetic and biophysical studies of the full‐length rat and human PAH (r/hPAH) homotetramers, there is consensus that this catalytic activation involves a slow (*s*‐to‐*min* timescale) global conformational change, preceding the chemical steps, characteristic of a hysteretic enzyme 2, 4. Mammalian PAHs are characterized by a complex substrate activation mechanism, and based on indirect experimental evidence two main working models have been proposed: (i) a binding of l‐Phe to a putative allosteric site in the N‐terminal autoregulatory domain as well as to the catalytic site 4, 5, 6, 7, 8, 9, and (ii) a cooperative binding of l‐Phe at the catalytic site (Hill coefficient *n*_H_ ~ 2.0) which represents the site of initiation (‘epicenter’) for the conformational transition in the activation process 10, 11, 12, 13, 14, 15. The first model was originally based on indirect enzyme kinetic 4 and biophysical studies on the rPAH homotetramer and truncated RD constructs, but has lately gained further support from the determination of th**e** high resolution crystal structure (PDB ID: 5FII at 1.8 Å) of a homodimeric truncated form of the human RD (hPAH‐RD) 16. Representing the key finding of this study, the structure revealed two l‐Phe molecules bound to a homodimer at the interface of the two β_1_α_1_β_2_β_3_α_2_β_4_ ACT domain folds along the plane of the twofold axis 16. Although the crystal structure of an enzyme·substrate complex in the full‐length homotetramer is still not available, our multiple crystal structures [see table in Ref. 17] of the catalytic CD (ΔN102/ΔC24‐hPAH‐Fe(III/II)), with different occupancy of the active site, are available in the PDB database 14, 18. They have revealed that binding of the pterin cofactor and/or substrates induce local and global conformational changes which are considered to be of functional importance, and leading to the proposal of a structure based reaction cycle for the hPAH‐CD enzyme 18. Spectroscopic analyses by magnetic circular dichroism and XAS of the full‐length hPAH homotetramer have supported this model 19.

In the Fe(II) form of the catalytic domain enzyme, the backbone of the partially intrinsically disordered Tyr^138^‐loop (residues Ala^132^‐His^146^) demonstrates a substrate induced conformational flexibility 18. It undergoes a refolding by hinge‐bending motions upon l‐Phe binding to the binary cofactor (BH_4_) complex, and a displacement of the C_α_‐atom of Tyr^138^ by ~ 10 Å from a surface position (Fig. 1A) to a largely buried position at the phenylalanine and cofactor binding pocket (Figs 1A,B and 6). However, in contrast to the catalytic domain, there is no crystal structure available for the substrate‐bound form of the full‐length homotetramer 9, 16. In our continuing effort to identify catalytically important residues, we have here addressed the functional role of this loop by point mutations of Tyr^138^, and compared the functional effects in the catalytic domain with the homotetrameric form of the human enzyme.

**Figure 1:**
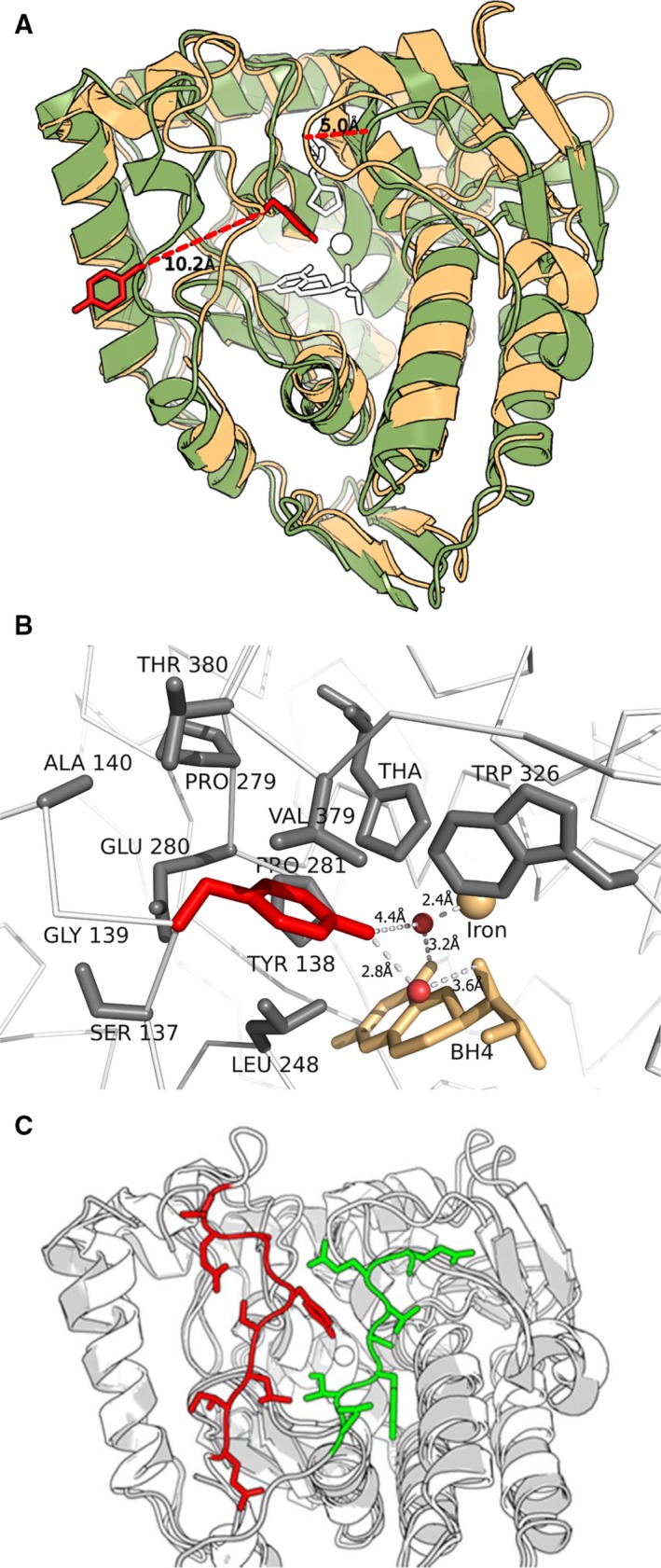
X‐ray crystal structures of hPAH forms used in this study. (A) Superimposition of the ternary complex of the catalytic domain ΔN102/ΔC24‐hPAH‐Fe(II)·BH_4_·3‐(2‐thienyl)‐l‐alanine (PDB ID: 1kw0, in pale red) onto the binary ΔN102/ΔC24‐hPAH‐Fe(II)·BH_4_ complex (PDB ID: 1j8u, in pale green) demonstrating the change in position for the flexible Tyr^138^‐loop (residues 132–146). Tyr^138^ is in stick model (dark red); the longest dotted line represents the Cα‐atom displacement of ~ 10 Å for Tyr^138^, bringing its hydroxyl group ~ 21 Å closer to the iron atom (Oη only 6.5 Å away). The substrate 3‐(2‐thienyl)‐l‐alanine and the pterin cofactor BH_4_ are shown in white stick model. The short dotted line represents the Cα‐atom displacement of ~ 5.0 Å for Thr^380^ in the mobile Thr^380^‐ῼloop. (B) Active site of hPAH. Shown are the residues with one atom within a distance of 5 Å from Tyr^138^ (in red) and 2 ordered water molecules (red dots). The distance between O in the –OH group and Wat O close to BH_4_ (biopterin) is 4.4 Å, and the distance from Wat O to O4 and N5 in BH_4_ is 3.2 and 2.6 Å, respectively. The figure was prepared using the atomic coordinates of PDB ID: 1kw0. (C) Superimposition of the crystal structure of the unliganded dimeric ΔC24‐rPAH‐Fe(III) (PDB IF: 1phz) onto the ternary complex of the catalytic domain ΔN102/ΔC24‐hPAH‐Fe(II)·BH_4_·3‐(2‐thienyl)‐L‐alanine (PDB ID: 1kw0) demonstrates that there is sufficient space for a displacement of the Tyr^138^‐loop (red) to the active site with the N‐terminal autoregulatory sequence (residues 19–33 green) in position. Close‐up view in which the Cα backbone of the flexible Tyr^138^‐loop (residues 134–139) and the Tyr^138^ side‐chain (red), and the N‐terminal autoregulatory sequence (green), are shown in stick model. The figures were created using pymol, version 1.7 31.

## Materials and methods

### Materials

The primers for site‐directed mutagenesis were obtained from Eurogentec (Seraing, Belgium) and MWG‐Biotech AG (Ebersberg, Germany). The QuikChange^®^ II site‐directed mutagenesis kit was from Stratagene (La Jolla, CA, USA). The BigDye^®^ Terminator v3.1 Cycle Sequencing Kit used to prepare DNA for sequencing was delivered by Applied Biosystems (Thermo Fisher Scientific Inc., Massachusetts, USA), and the DNA sequencing was carried out on an ABI 3730xl DNA Analyzer (Applied Biosystems). Factor Xa was obtained from Protein Engineering ApS (Aarhus, Denmark). The reagents and the sensor chip CM5 used in the surface plasmon resonance (SPR) analyses were purchased from GE Healthcare Life Science (Uppsala, Sweden). (6*R*)‐L‐*erythro*‐5,6,7,8‐tetrahydrobiopterin (BH_4_) was delivered by Dr. B. Schircks Laboratory (Jona, Switzerland).

### Generation of mutational variants

The substitutions (Phe, Ala, Lys and Glu) were introduced into the cDNAs of the wt‐hPAH and the double truncated form ΔN102/ΔC24‐hPAH (catalytic domain) using the QuikChange^®^ II site‐directed mutagenesis kit (Thermo Fisher Scientific Inc.). The pMAL‐hPAH 20 and pMAL‐ΔN102/ΔC24‐hPAH 21 plasmids, containing a cleavage site for factor Xa, were used as template, and the specific oligonucleotide primers listed in Table S1 were used for mutagenesis.

### Expression and purification of enzyme proteins

The wt‐hPAH, ΔN102/ΔC24‐hPAH and their respective mutant fusion proteins were expressed in *Escherichia coli* (TB1 cells) with maltose‐binding protein (MBP) as fusion partner. The bacteria were harvested after 8 h of induction with 1 mm isopropyl‐thio‐β‐d‐galactoside at 28 °C, and the tetrameric and dimeric fusion proteins were purified by affinity chromatography and size‐exclusion chromatography (SEC) to homogeneity as described 22. The fusion proteins were cleaved by factor Xa and followed by SEC as described 22. The isolated tag‐free wt‐hPAH tetramers and ΔN102/ΔC24‐hPAH dimers (and the corresponding mutant proteins) were collected and concentrated 22. Protein concentration was determined using the absorption coefficient A280 (1 mg·mL^−1^·cm^−1^) of 1.0 for the full‐length wt‐hPAH 22 and of 1.24 for the truncated ΔN102/ΔC24‐hPAH, according to the method of Gill and von Hippel 23 in 20 mm Na‐Hepes, 200 mm NaCl, pH 7.0, with and without 6 m guanidine chloride. The same method was used for the isolated Tyr^138^→Phe/Ala/Lys/Glu full‐length mutants, A_280_ = 0.92, and for the isolated Tyr^138^→ Phe/Ala/Lys/Glu ΔN102/ΔC24‐hPAH, A_280_ = 1.19. Protein purity was analyzed by SDS/PAGE in a 10% (w/v) polyacrylamide gel 24. The gels were stained by Coomassie Brilliant Blue R‐250, scanned using VersaDoc 4000 (Bio‐Rad, Hercules, CA, USA) and quantification of the protein bands was obtained by using the quantity one 1‐d analysis Software (Bio‐Rad).

### Assay of enzymatic activity and coupling efficiency

The catalytic activity was assayed at 25 °C in a medium containing 100 mm Na‐Hepes (pH 7.0), 5 mm DTT, 0.04 mg·mL^−1^ catalase, 10 μm ferrous ammonium sulfate, 0.5 mg·mL^−1^ bovine serum albumin, 0.3−0.9 μm subunit of hPAH tetramer or ΔN102/ΔC24‐hPAH dimer and variable concentrations of l‐Phe and pterin cofactor (BH_4_). After 5‐min preincubation with l‐Phe, the reaction was initiated by adding BH_4_ with DTT, and allowed to proceed as described 25. The amount of L‐Tyr formed after one minute (standard), or other selected time points, was measured by HPLC with fluorimetric detection 22. The steady‐state kinetic parameters were calculated by non‐linear regression analysis using the sigmaplot ^®^ Technical Graphing Software (Allsoft AS, Lillestrøm, Norway) and the modified Hill equation of LiCata and Allewell 26 for cooperative substrate binding as well as substrate inhibition 25, i.e. the velocity *ν* = {*V*_max_ + *V*_i_([*S*]^x^/*K* ^x^_i_)}/{1 + (*K* ^h^/[*S*]^h^) + ([*S*]^x^/*K* ^x^_i_)} 26. The exponent *x* is a second Hill coefficient which allows for the possibility that the substrate inhibition may also be cooperative, and by varying the value of *x* between 1 and 3, *x* = 2 gave the best fit for our values of the wt full‐length enzyme. [*S*]_0.5_ is taken as the concentration of substrate at one‐half the calculated *V*_max_. In order to study the effect of preincubation with l‐Phe on the specific activity (fold activation), 1 mm l‐Phe was added either at the start of the preincubation period or together with 75 μm BH_4_ at the initiation of the reaction. The coupling efficiency 27 of the hydroxylation reaction was measured in a mixture containing 0.3−1.5 μm hPAH, 200 μm NADH, 0.05 μg·μL^−1^ catalase, 10 units superoxide dismutase, 10 μm ferrous ammonium sulfate, 1 mm l‐Phe, excess dihydropteridine reductase in 187 mm Hepes buffer, pH 7 at 25 °C. The reaction was started by adding 100 μm BH_4_, and the oxidation of NADH was followed in real‐time at 340 nm, using an Agilent 8453 Diode Array spectrophotometer with a Peltier temperature control unit. At selected time points aliquots of the reaction mixture were mixed with acidic ethanol (stop solution), and the amount of l‐Tyr formed was measured by HPLC with fluorimetric detection 22. Coupling efficiencies were calculated and defined as the rate of l‐Tyr formation divided by the rate of NADH consumption using the molar extinction coefficient ε = 6220 m ^−1^·cm^−1^ for the coenzyme 27. Quantitative data are presented as mean ± SD of three to six independent assays.

### Conformational analysis in real time by SPR spectroscopy

The l‐Phe‐induced conformational change (hysteresis) of full‐length hPAH homotetramers, including a reported increase in the hydrodynamic radius and volume (~ 10%) 1, was measured by real‐time SPR spectroscopy with isolated tetramers as described 28 using the Biacore 3000 biosensor system (GE Healthcare Life Science). The full‐length wt and mutant tetramers, diluted in 10 mm sodium acetate buffer (pH 5.5) to a final concentration of 0.23 mg·mL^−1^, were immobilized covalently to the hydrophilic carboxymethylated dextran matrix CM5 sensor chip by the primary amine coupling reaction. Due to its low molecular mass the analyte l‐Phe (165 Da) is SPR transparent. Since the ligand‐free catalytic domain enzyme is already in an activated state (Table 1), it did not demonstrate any time‐dependent response to l‐Phe binding, but only a minor square‐wave SPR signal increase ~ 0.04 RU·ng protein^−1^·nm^−2^ (Fig. S1C). This protein was therefore immobilized in the reference channel in all the analyses of the full‐length enzymes. In the enzyme homotetramer the equilibrium response was reached after about 3 min, and representative examples for different molecular forms are shown in Fig. S1A,B. The equilibrium responses (ΔRU_eq_ at *t* = 3 min) as a function of the free l‐Phe concentration were used to determine the concentration at half maximal response ([*L*]_0.5_) and the maximum ΔRU_eq_ value by nonlinear regression analysis using the sigmaplot ^®^ Technical Graphing Software. The experimental error for replicate injections of the analyte was < 4%. The SPR responses were expressed as ΔRU (ng protein·mm^−2^)^−1^ where 1000 RU corresponds to ~ 1 ng immobilized protein·mm^−2^ 29.

**Table 1:** Steady‐state kinetic properties of the dimeric double truncated form ΔN102/ΔC24‐hPAH and its Y138X mutants, and the tetrameric wild‐type hPAH and its Y138X mutants. The kinetic properties and coupling efficiencies were determined at 25 °C; the substrate concentrations were 1 mm l‐Phe (BH_4_ variable) and 75 or 100 μm BH_4_ (l‐Phe variable)

hPAH	l‐Phe						BH_4_	
	*V*_max_ (nmol Tyr·min^−1^·mg^−1^)	[*S*]_0.5_ (μm)	*n*_*H*_	*k*_cat_/[*S*]_0.5_ a (μm ^−1^·min^−1^)	Substrate inhibition	Fold activation	*V*_max_ (nmol Tyr·min^−1^·mg^−1^)	*K*_m_ (μm)
ΔN102/ΔC24	7948 ± 339	46 ± 4	~ 1^b^	6.50	Yes (pronounced)	0.9 ± 0.1	4804 ± 222	25 ± 4
ΔN102/ΔC24‐Y138F	4995 ± 346	47 ± 6	~ 1^b^	4.00	Yes (pronounced)	0.9 ± 0.1	4638 ± 214	62 ± 7
ΔN102/ΔC24‐Y138A	2385 ± 118	36 ± 3	~ 1^b^	2.49	Yes (pronounced)	0.8 ± 0.1	1267 ± 70	27 ± 5
ΔN102/ΔC24‐Y138E	2068 ± 70	63 ± 4	~ 1^b^	1.23	Yes	1.0 ± 0.1	2336 ± 94	60 ± 6
ΔN102/ΔC24‐Y138K	2174 ± 53	128 ± 7	~ 1^b^	0.64	Yes	0.9 ± 0.1	2384 ± 73	58 ± 4
Wild‐type	5056 ± 222	170 ± 10	1.95 ± 0.17	1.49	Yes	5.3 ± 0.5	5840 ± 262	39 ± 5
Y138F	2869 ± 145	172 ± 7	1.70 ± 0.11	0.83	Yes	5.2 ± 0.5	1939 ± 83	35 ± 5
Y138A	1653 ± 57	144 ± 9	1.80 ± 0.15	0.57	Yes	9.2 ± 0.5	1924 ± 119	47 ± 8
Y138E	856 ± 13	203 ± 9	1.47 ± 0.06	0.21	No	2.2 ± 0.2	752 ± 19	32 ± 3
Y138K	702 ± 16	459 ± 27	1.20 ± 0.07	0.08	No	3.9 ± 0.3	668 ± 29	44 ± 5

a The catalytic efficiency was calculated on the basis of a subunit molecular mass of 50 kDa for the full‐length forms and 37.6 kDa for the double truncated forms of hPAH. [*S*]_0.5_ represents the l‐Phe concentration at half‐maximal activity and *k*_cat_/[*S*]_0.5_ is here defined as the catalytic efficiency.

b The calculation of a reliable Hill coefficient (*n*_H_) using all the data points (up to 4.0 mm l‐Phe) is complicated by the pronounced substrate inhibition (see Fig. 2 and the main text), but using only data points ≤ 500 μm a value of unity was calculated for *n*_H,_ in agreement with previous studies 21.

### Structural bioinformatic analyses

To identify the location of potential hinge‐bending regions in PAH we subjected the coordinates of unliganded and non‐phosphorylated rPAH RD+CD (PDB ID: 2phm) to further analysis using the hingemaster software program that predicts the location of hinges in a protein by integrating existing hinge predictors (TLSMD, StoneHinge, FlexOracle and HingeSeq) with a family of hinge predictors based on grouping residues with correlated normal mode motions 30. This truncated form was selected due to its higher resolution than the full‐length tetramers. 3D structural images were made in the software pymol, version 1.7 31.

## Results

The isolated MBP fusion proteins of ΔN102/ΔC24‐hPAH and wt‐hPAH, and their mutant forms, were cleaved with factor Xa, and the subsequent SEC chromatography resulted in comparable high yields (mg quantities) of soluble homooligomeric enzyme forms. As expected 21, 22, the full‐length forms exists in an equilibrium of predominantly tetramers (~ 209 kDa) and some dimers (~ 104 kDa), and the catalytic core enzymes (ΔN102/ΔC24‐hPAH) were recovered as dimers (~ 70 kDa) (SEC data not shown). The protomers for the wt and mutant full‐length tetrameric forms revealed identical electrophoretic mobilities on SDS/PAGE (Fig. S2A,B). For the wt and mutant catalytic domain enzymes two trace contaminant proteins (< 5% of total protein) were observed.

### The effect of Tyr^138^ substitutions on the catalytic activity and coupling efficiency

The catalytic domain dimer, in its non‐mutated form, revealed a fourfold higher catalytic efficiency (*k*_cat_/[*S*]_0.5,_ _l__‐Phe_) than the l‐Phe activated full‐length wt tetramer, and the former was not further activated by preincubation with substrate (Fig. 2 and Table 1). Both enzyme forms demonstrated substrate inhibition (Fig. 2), that was very pronounced for the non‐mutated and mutant catalytic domain dimers, which prevented the determination of reliable Hill coefficients for these forms when all the data points were used (see footnote in Table 1). A high linear correlation (*r* ^2^ = 0.99) was observed between the effects of the four substitutions on the catalytic efficiency of the two molecular forms (Fig. 3). All the mutant tetramers revealed a reduced kinetic cooperativity (Table 1), most pronounced for the Glu^138^ and Lys^138^ mutant forms, and a slight (8–22%) ‘loose’ coupling was also observed (Table 2).

**Figure 2:**
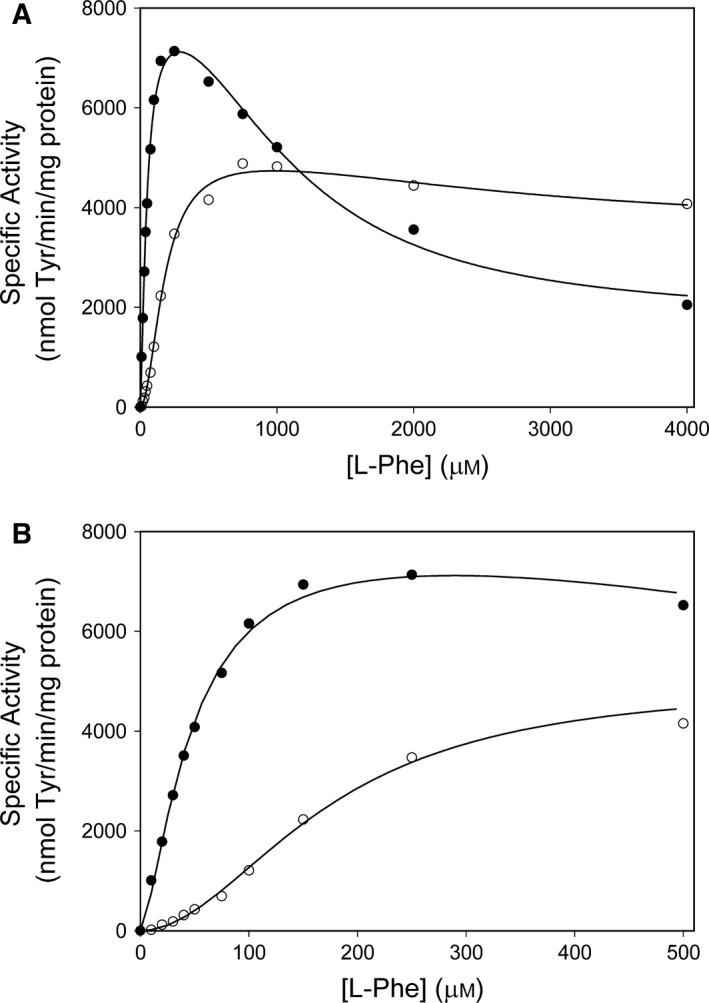
The effect of l‐Phe concentration on the catalytic activity of the catalytic domain ΔN102/ΔC24‐hPAH dimer (●) and of the full‐length wt‐hPAH tetramer (○). (A) The activity was assayed at standard assay conditions (0–4 mm l‐Phe, 75 μm BH_4_ and 25 °C). (B) Close‐up of the data shown in (A) for the concentration range 0–500 μm l‐Phe, demonstrating a positive kinetic cooperativity for the full‐length wt‐hPAH tetramer.

**Figure 3:**
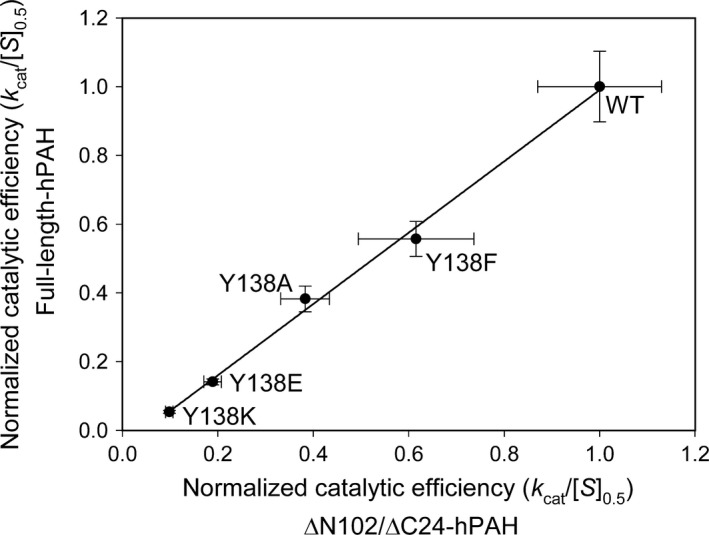
The effect of Tyr^138^ substitutions on the catalytic efficiency of hPAH. Secondary plot demonstrating the positive correlation (*r* ^2^ = 0.99) between the catalytic efficiency (*k*_cat_/[*S*]_0.5_) of the Tyr^138^Phe/Ala/Glu/Lys mutations in the catalytic domain (ΔN102/ΔC24‐hPAH) dimer and the l‐Phe activated full‐length enzyme tetramer. The primary data are given in Table 1. The values for the catalytic efficiency were normalized with the wt full‐length and wt catalytic CD as a reference (1.0). Error bars represent SD.

**Table 2:** The degree of coupling of the hydroxylation reaction catalyzed by the dimeric double truncated form ΔN102/ΔC24‐hPAH and its Y138X mutant, and tetrameric wild‐type hPAH and its mutants. The data represent the mean values of three to six independent assays

hPAH	Coupling efficiency (mol Tyr formed/mol BH_4_ oxidized)
ΔN102/ΔC24	0.97 ± 0.07
ΔN102/ΔC24‐Y138F	1.08 ± 0.05
ΔN102/ΔC24‐Y138A	0.96 ± 0.05
ΔN102/ΔC24‐Y138E	1.03 ± 0.02
ΔN102/ΔC24‐Y138K	1.00 ± 0.02
Wild‐type	0.97 ± 0.03
Y138F	0.92 ± 0.03
Y138A	0.82 ± 0.11
Y138E	0.78 ± 0.07
Y138K	0.87 ± 0.05

### The Tyr^138^ substitutions perturb substrate activation of the full‐length tetramer and related conformational changes

The wt full‐length tetramer is activated several‐fold by preincubation with the substrate, and displays a positive kinetic and binding cooperativity with respect to l‐Phe (for review, see 1, 2, 3, 4). Here, the wt‐hPAH tetramer revealed a kinetic Hill coefficient of *n*_H_ = 1.95 ± 0.17, a [*S*]_0.5_‐value of 170 ± 10 μm l‐Phe and a 5.3‐fold (± 0.5) enhancement of the catalytic activity (activation) on preincubation (5 min) with 1 mm l‐Phe at 25 °C (Table 1). Interestingly, the Ala^138^ mutant tetramer revealed a slightly decreased [*S*]_0.5_‐value (144 ± 9 μm), and a higher degree, i.e. 9.2‐fold (± 0.5) of catalytic activation by 1 mm l‐Phe. Removing the hydroxyl group (Phe^138^ substitution) has no significant effect on fold‐activation, whereas the Glu^138^ and Lys^138^ substitutions demonstrate more pronounced perturbing effects (Table 1), presumably related to an unfavorable polarity/charge and side‐chain volume/steric effect in the interaction with the protein in general.

**Figure 4:**
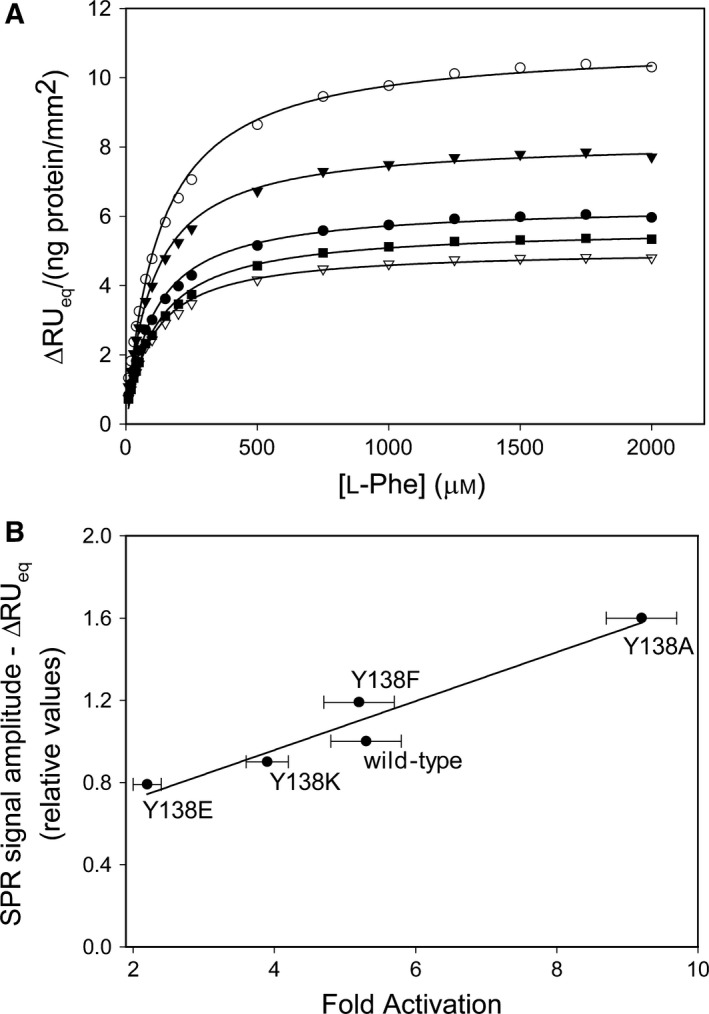
The effect of Tyr^138^ substitutions on the conformational isomerization (hysteresis) of hPAH. (A) The SPR equilibrium response (ΔRU_eq_) isotherm for immobilized full‐length wt‐hPAH tetramer and the Tyr^138^Xxx mutant forms as a function of the l‐Phe concentration; wt‐hPAH (●), Tyr^138^Phe‐hPAH (▼), Tyr^138^Ala‐hPAH (○), Tyr^138^Glu‐hPAH (▽) and Tyr^138^Lys‐hPAH (■). The non‐cooperative isotherm for wt‐hPAH gave an [*L*]_0.5_ value of 97 ± 6 μm l‐Phe. The catalytic domain enzyme ΔN102/ΔC24‐hPAH was immobilized to the reference cell to subtract any contribution of l‐Phe binding. The experiments were performed at 25 °C in HBS‐EP buffer (10 mm Hepes, 150 mm NaCl, 3.4 mm EDTA and 0.005% (v/v) of the surfactant P20, pH 7.4). The experimental error for replicate injections of the analyte was < 4%. The curve fitting of the response isotherms was obtained by non‐linear regression analysis using the sigmaplot ^®^ Technical Graphing Software with an *r* ^2^ = 0.99. (B) Secondary plot demonstrating the relationship between the l‐Phe‐induced fold activation and ΔRU_eq_ of full‐length wt‐hPAH tetramer and the Tyr^138^Xxx mutant forms. The fold activation values are given in Table 1. The SPR values represent the equilibrium response at 1 mm l‐Phe calculated from the respective response isotherms in (B) (each based on *n* = 19 individual l‐Phe concentrations), and is given as relative values with wt as a refrence (1.0). A strong positive correlation (*r* ^2^ = 0.96) was observed between the two parameters.

When the tetramer binds l‐Phe, the enzyme undergoes local and global conformational changes, including a reported increase in hydrodynamic radius and volume (volume ~ 10% for rPAH) 1, and it is catalytically activated. Intrinsic tryptophan fluorescence 4, 28 and SPR spectroscopy 28 have been used to monitor in real time two different manifestations of this process, both on a *s*‐to‐*min* time scale. Here, the full‐length wt tetramer and its mutant forms were immobilized to the dextran matrix of the CM5 sensor chip 28. From the SPR analysis a typical time‐dependent increase in the signal response to l‐Phe injection was observed for the wt‐hPAH tetramer, and all the Tyr^138^ substitutions, with ΔRU_eq_ values reached at ~ 3 min (Fig. 4A). The mutant forms revealed a variable ΔRU_max_ value, calculated by non‐linear regression analyses of the l‐Phe *versus* ΔRU_eq_ response isotherms, based on 17 individually obtained data points (Fig. 4A). For the wt tetramer an [*L*]_0.5‐_value of 97 ± 6 μm for l‐Phe was calculated. Whereas ΔRU_max_ (at 2.0 mm l‐Phe) increased from ~ 5.9 RU·ng protein^−1^ mm^−2^ in the wt to ~ 7.7 RU·ng protein^−1^ mm^−2^ in the Phe^138^ substitution and to ~ 10.2 RU·ng protein^−1^ mm^−2^ in the Ala^138^ substitution, it was reduced in the Lys^138^ and Glu^138^ substitutions. Moreover, the fold increase in catalytic activity, on preincubation with 1 mm l‐Phe (fold activation) (Table 1), revealed a high linear correlation (*r* ^2^ = 0.96) to the ΔRU_eq_ value of the SPR signal response at the same l‐Phe concentration (Fig. 4B).

### *In silico* prediction of hinge‐bending regions

**Figure 5:**
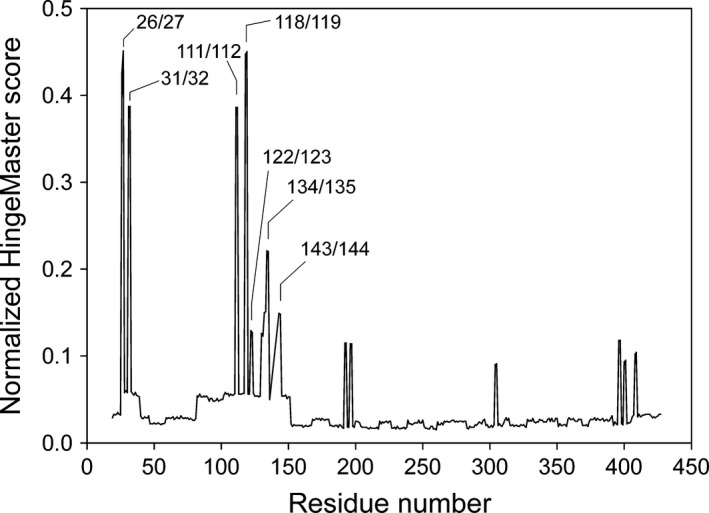
*In silico* analyses of truncated forms of hPAH. Combined and normalized hinge scores for the C‐terminal truncated rPAH (ΔC24‐rPAH‐Fe(III)) (PDB ID 2phm), using the HingeMaster server 30. Potential hinge bending regions with signed peak residues, that promote intrasubunit communication, are indicated.

Using the atomic coordinates of the nonphosphorylated ΔC24‐rPAH‐Fe(III) enzyme (PDB ID: 2phm at 2.6 Å), the hingemaster program predicted, with a high score, two hinge regions within the Y^138^‐loop (at Q^134^‐I^135^ and D^143^‐A^144^) and two at the interface of the regulatory and catalytic domain (at R^111^‐D^112^ and V^118^‐P^119^) as well as two regions within the N‐terminal tail (at E^26^‐D^27^ and Q^31^‐N^32^) (Fig. 5).

## Discussion

Enzyme catalysis is an inherently dynamic process and the existence of motions on various time scales is well accepted, but so far a limited number of enzymes have been shown to rely on essential coupled residue motions for catalysis, e.g. on loop motions (for review see 32). Here we have addressed the functional importance of the partially intrinsically disordered and highly conserved Tyr^138^‐loop in the full‐length hPAH homotetramer 9, which was seen to have missing electron densities of the residues S^137^YGAEL in the unliganded rPAH tetramer 19. In the catalytic domain structure, where the electron density of the whole loop is preserved, the loop also demonstrates a conformational flexibility, but here with a more ordered structure 14, 18.

### Loop motions and conformational dynamics related to substrate binding as determined by crystallography

**Figure 6:**
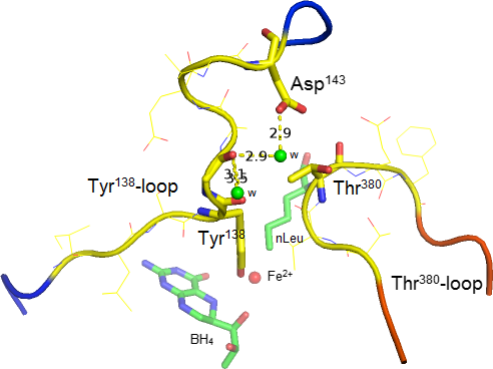
The close association of the mobile Tyr^138^‐loop and the mobile Thr^380^‐ῼloop in the X‐ray crystal structure of the ternary complex of the hPAH catalytic domain ΔN102/ΔC24‐hPAH‐Fe(II)·BH_4_·norleucine (PDB ID: 1mmt, at 2.0 Å resolution). The loop‐loop interaction is defined by the closest distance of ~ 3.5 Å between the main‐chain carbonyl oxygen of Tyr^138^ and the side‐chain of Thr^380^. Together the two loops fold over the active site like a lid domain. The Tyr^138^‐loop is stabilized by hydrogen bonds to three structural water molecules (green spheres).

The catalytic domain structures provide the first insight into what might be part of the catalytic mechanism of the full‐length enzyme tetramer. In the catalytic domain for the hPAH enzyme, the binding of BH_4_ cofactor causes structural changes at its active site and conformational changes at the Thr^380^‐ῼloop (Q^375^EYSVTEFQPL) 33, 34. Subsequent binding of substrate analogs triggers a change in the coordination (from six to five) of the catalytic iron (Fe(II)), a motion of BH_4_ and the residues lining the active site crevice are rearranged 18, 34. Moreover, the Tyr^138^‐loop is refolded (Fig. 1A), bringing the Tyr^138^‐OH group ~ 21 Å closer to the iron (Oƞ atom only ~ 6.5 Å away, Fig. 1B) 18. *In silico* two hinge regions are predicted within the loop, at residues Gln^134^‐Ile^135^ and Asp^143^‐Ala^144^. These residues are highly accessible to the solvent and feature a low number of atomic interactions with the rest of the protein, which may facilitate hinge motion. In the crystal structure the loop is stabilized by hydrogen bonds mediated by three water molecules (Fig. 6), where the one to the side‐chain of Asp^143^ is of notable interest (see Discussion below). The binding of the l‐Phe analog 3‐(2‐thienyl)‐l‐alanine (Fig. 1A,B) or norleucine (Fig. 6) is also associated with a motion of the smaller interacting Thr^380^‐ῼloop in which the C_α_ atom of Thr^380^ moves ~ 5.0 Å in the direction of the Tyr^138^‐loop (Fig. 1A). Together the two loops fold over the active site like a lid domain (Fig. 6), and allows the enzyme to interconvert between an open and closed conformation, from which catalysis occurs 18. The observed high degree of coupling efficiency of 1.0 in the catalytic reaction (Table 2) may be explained by an effective protection of the cofactor. The loop‐loop interaction in the ternary complex is defined by the closest distance of ~ 3.5 Å between the main‐chain carbonyl oxygen of Tyr^138^ and the side‐chain of Thr^380^ (Fig. 6), reduced from 15.9 Å in the BH_4_ binary complex with cofactor 34. The X‐ray structure of the inactive unliganded form of the full‐length rPAH tetramer demonstrates a similar relative position of the two loops, but with a missing electron density for residues 137–142. Unfortunately, no crystal structure is yet available for this enzyme form with bound substrate and/or cofactor, but some information has been reported on its solution structure based on SAXS analyses of hPAH in the absence and presence of l‐Phe 9.

### Loop mutations perturb the catalytic and coupling efficiency as well as substrate activation and related conformational changes

In the catalytic domain the Tyr^138^‐loop is a functional loop and its motion is triggered by l‐Phe (or analog) binding at the active site 14, 18. The multiple substitutions of Tyr^138^ differentially perturb functional properties of this enzyme dimer and the full‐length tetramer. They both reduce the *V*_max_ values for tyrosine synthesis, and the catalytic efficiency (*k*_cat_/[*S*]_0.5_) was equally reduced (*r* ^2^ = 0.99 in the two enzyme forms (Table 1 and Fig. 3). In the full‐length enzyme the coupling efficiencies were in addition partly (8–22%) reduced (Table 2). An interesting outcome of the present study is the demonstration that the loop and the residue Tyr^138^ play a role also in the l‐Phe‐induced substrate activation and related hysteretic conformational changes (SPR), which are linearly correlated (*r* ^2^ = 0.96) (Fig. 4B). Our results underline that protein motion and catalytic activity are coupled, and that the Tyr^138^‐loop represents a key mediator in coupling protein motion with enzyme function. The substitutions of Tyr^138^ may perturb the normal gating mechanism in the refolding of the Tyr^138^‐loop.

An interesting variant is the Ala^138^ substitution in both enzyme forms. It is characterized by a reduced value for [*S*]_0.5,_ _l__‐Phe_ (Table 1) and no significant change in *K*_m,BH4_, but a ~ 60% reduction in catalytic efficiency (*k*_cat_/[*S*]_0.5_), and a slight ‘loose’ coupling (~ 20%) of the reaction in the full‐length tetramer (Table 2). Moreover, l‐Phe triggers a proportional increase in the catalytic activation (from 5.3‐fold in Tyr^138^‐PAH to 9.2‐fold in Ala^138^‐PAH) and the SPR signal response, related to the global conformational change (Fig. 4B). The effects indicate that the substitution with a smaller and apolar side‐chain may facilitate loop/hinge motions, i.e. the gating of the surface located Ala^138^‐loop toward the active site, which is structurally characterized by a network of hydrophobic interactions 14, 18, 34. But the cofactor BH_4_ or its catalytic intermediates at the active site is partly destabilized, as indicated by the slight degree of ‘loose’ coupling. Thus, the substrate seems to stabilize the enzyme in the binary complex, but not in the ternary complex, possibly by pushing the conformational equilibrium away from the active form. The moderate functional effects of the Tyr^138^ substitutions may also be explained by a motion of the Thr^380^‐ῼloop upon substrate binding, independent of the mutations in the other loop, since it is part of the active site lid (Figs 1A and 6).

### From catalytic domain dimer to full‐length PAH homotetramer

Our Tyr^138^‐loop mutation analyses, demonstrating a high linear correlation (*r* ^2^ = 0.99) in perturbing the catalytic efficiency of the two enzyme forms (Fig. 3), support the conclusion that the dynamics and function of the Tyr^138^‐loop obtained for the dimeric catalytic domain enzyme is representative for the full‐length enzyme tetramer. The functional importance of the loop in the wt enzyme homotetramer is further underlined by three PKU and loop related mutations. In particular the mutation p.Asp^143^Gly which is located at the predicted hinge region Asp^143^‐Ala^144^ (Fig. 5). As indicated in Fig. 6 the position of Asp^143^ is stabilized by a hydrogen bond to a water molecule, and the substitution with a Gly residue is expected to affect the loop mobility, and thus have an effect on the active site. When expressed in three different systems (*E. coli*, 293‐cells and an *in vitro* transcription‐translation system), the recombinant mutant enzyme represents a kinetic variant form, in which both the substrate and cofactor are bound with reduced affinity 35. Moreover, a high degree of similarity exists between the truncated dimeric form ΔC24‐rPAH (PDB ID: 1phz at 2.2 Å resolution) 5 and the full‐length unliganded and inactive rPAH tetramer (PDB ID: 5den at 2.9 Å) 16 in terms of r.m.s. deviation, including the loops. Based on its atomic coordinates, *in silico* analyses (Fig. 5) predict two putative hinge‐bending regions in the Tyr^138^‐loop, at residues Gln^134^‐Ile^135^ and Asp^143^‐Ala^144^. The predicted motion of the loop corresponds to that actually observed crystallographically for the hPAH catalytic domain 14, 18. But in contrast to this enzyme form there is no crystal structure yet available for the substrate‐bound mammalian enzyme tetramer. Interestingly, superimposition of the ternary complex of the hPAH catalytic domain (PDB ID: 1kw0) onto the structure of the unliganded (regulatory + catalytic) domain rPAH enzyme (PDB ID: 1phz) demonstrates that there is sufficient space for a displacement of the Tyr^138^‐loop in direction of the active site with the N‐terminal autoregulatory sequence (residues 19–33) in position (Fig. 1C).

## Conclusion

The regional flexibility/motion of the Tyr^138^‐loop is a key element in the biological function of mammalian PAH, whose conformation and function is triggered by the binding of its substrate. l‐Phe binding at the active site is accompanied by structural rearrangements at that site in addition to its effect on loop dynamics. Based on previous crystallographic and structural modeling and the current site‐directed mutagenesis, it may be inferred that the functional information obtained about the Tyr^138^‐loop in the catalytic domain enzyme, can be extended to include the full‐length enzyme homotetramer.

## Author contributions

JL, AJS and TF designed the study; JL and AJS carried out the main experiments; JL, AJS, KT and TF analysed the data and wrote the manuscript. All authors have read and approved the manuscript.

## Supporting information


**Fig. S1.** Representative sensorgrams for the binding of l‐Phe to different molecular forms of hPAH as determined by SPR analyses.
**Fig. S2.** SDS/PAGE analysis of the purified recombinant hPAH proteins.
**Table S1.** Oligonucleotides used for site‐directed mutagenesis.
