# Supplementary material for: Substituting Tyr138 in the active site loop of human phenylalanine hydroxylase affects catalysis and substrate activation
Source: FEBS Open Bio. 2017 Jun 12;7(7):1026–36. doi: 10.1002/2211-5463.12243 (PMC5494296; doi:10.1002/2211-5463.12243)
Supplement: Supplementary file 1 — Fig. S1. Representative sensorgrams for the binding of l‐Phe to different molecular forms of hPAH as determined by SPR analyses. Fig. S2. SDS/PAGE analysis of the purified recombinant hPAH proteins. Table S1. Oligonucleotides used for site‐directed mutagenesis. [file FEB4-7-1026-s001.doc]

*Supplementary data* *for*

**Substituting Tyr^138^ in the active site loop affects catalysis and substrate activation in human phenylalanine hydroxylase**

João Leandro^1,2#^, Anne J. Stokka^1,3^, Knut Teigen^1^, Ole A. Andersen^1,4^ and Torgeir Flatmark^1^

^1^ *Department of Biomedicine, University of Bergen, Jonas Lies vei 91, N-5009 Bergen, Norway*

^2^ *Metabolism and Genetics Group, Research Institute for Medicines (iMed.ULisboa), Faculty of Pharmacy, University of Lisbon, Av. Prof. Gama Pinto, 1649-003 Lisbon, Portugal*

^3^ *The Biotechnology Centre of Oslo, University of Oslo, Gaustadallèen 21, N-0349 Oslo, Norway*

^4^ *Evotec (UK) Ltd., 114 Innovation Drive, Milton Park, Abingdon, OX14 4RZ, UK*

^#^ Current address: Department of Genetics and Genomic Sciences, Icahn School of Medicine at Mount Sinai, 1425 Madison Avenue, Box 1498, New York, NY 10029, USA

Running Title: Functional role of the Tyr^138^-loop in human phenylalanine hydroxylase

**Correspondence**

T. Flatmark, Department of Biomedicine, University of Bergen, Jonas Lies vei 91, N-5009 Bergen, Norway

Fax: +47 55586360

Tel: +47 55586428

E-mail: [torgeir.flatmark@biomed.uib.no](mailto:torgeir.flatmark@biomed.uib.no)

**Supplementary Fig. S1**


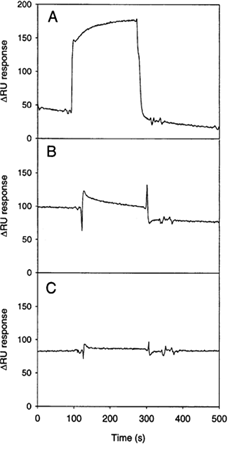


**Supplementary Fig. S1.** **Representative sensorgrams for the binding of L-Phe to different molecular forms of hPAH as determined by SPR analyses.** The sensorgrams display the reversible increase in refractive index induced by the binding of L-Phe (*A*) to tetrameric wt-hPAH, (*B*) to the N-terminal truncated form ΔN(1-102)-hPAH, and (*C*) to the double truncated form ΔN(1-102)/ΔC(428-452)-hPAH. L-Phe (2 mM) in HBS buffer was injected over the immobilized enzyme forms. Immobilization of wt-hPAH resulted in a SPR response of 26500 RU, consistent with 0.127 pmol enzyme/mm^2^ (0.51 pmol subunit/mm^2^); immobilization of ΔN(1-102)-hPAH resulted in a response of 17300 RU, consistent with 0.108 pmol enzyme/mm^2^ (0.432 pmol subunit/mm^2^) and immobilization of ΔN(1-102)/ΔC(428- 452)-hPAH resulted in a response of 14980 RU, consistent with 0.192 pmol enzyme/mm^2^ (0.384 pmol subunit/mm^2^). For calculations, see the Materials and methods section. In all three experiments immobilized lysozyme (8300 RU) was present in the reference cell.

**Supplementary Fig. S2**

**Supplementary Fig. S2.** **SDS-PAGE analysis of the purified recombinant hPAH proteins.** (*A*) Full-length tetrameric WT-hPAH and respective tetrameric mutant forms. *Lane* 1, WT-hPAH; *lane* 2, Y138F-hPAH; *lane* 3, Y138A-hPAH; *lane* 4, Y138E-hPAH; *lane* 4, Y138K-hPAH. (*B*) Double truncated dimeric catalytic core ΔN102/ΔC24-hPAH and respective double truncated dimeric mutant forms. *Lane* 1, low-molecular-mass standard; *lane* 2, ΔN102/Δ24-hPAH; *lane* 3, ΔN102/ΔC24-hPAH-Y138F; *lane* 4, ΔN102/ΔC24-hPAH-Y138A; *lane* 5, ΔN102/ΔC24-hPAH-Y138E; *lane* 6, ΔN102/ΔC24-hPAH-Y138K.

**Supplementary Table S1.**

Oligonucleotides used for site-directed mutagenesis. Mismatch nucleotides are shown in boldface.

| Primer | Sense | PAH cDNA position^a^ | Sequence (5ʹ→3ʹ) |
| --- | --- | --- | --- |
| Y138F | Forward | 628-648 | CTCAGCT**T**TGGAGCGGAACTG |
| Y138F | Reverse | 628-648 | CAGTTCCGCTCCA**A**AGCTGAG |
| Y138A | Forward | 628-648 | CTCAGC**GC**TGGAGCGGAACTG |
| Y138A | Reverse | 628-648 | CAGTTCCGCTCCA**GC**GCTGAG |
| Y138K | Forward | 622-653 | CAGATTCTCAGC**A**A**A**GGAGCGGAACTGGATGC |
| Y138K | Reverse | 622-653 | GCATCCAGTTCCGCTCC**T**T**T**GCTGAGAATCTG |
| Y138E | Forward | 622-653 | CAGATTCTCAGC**G**A**G**GGAGCGGAACTGGATGC |
| Y138E | Reverse | 622-653 | GCATCCAGTTCCGCTCC**C**T**C**GCTGAGAATCTG |

^a^Data are taken from [1].

**Supplementary References**

1. Kwok, S.C., Ledley, F.D., DiLella, A.G., Robson, K.J., Woo, S.L. (1985) Nucleotide sequence of a full-length complementary DNA clone and amino acid sequence of human phenylalanine hydroxylase. *Biochemistry* **24**, 556-561
